# The Role of Aortic Volume in the Natural History of Abdominal Aortic Aneurysms and Post-Endovascular Aortic Aneurysm Repair Surveillance

**DOI:** 10.3390/jcm13010193

**Published:** 2023-12-29

**Authors:** George Kouvelos, George Volakakis, Konstantinos Dakis, Konstantinos Spanos, Athanasios Giannoukas

**Affiliations:** Department of Vascular Surgery, University Hospital of Larissa, Faculty of Medicine, School of Health Sciences, University of Thessaly, 41110 Larissa, Greece; volakaki@med.uth.gr (G.V.); kostasdakis1994@gmail.com (K.D.); spanos.kon@gmail.com (K.S.); agiannoukas@hotmail.com (A.G.)

**Keywords:** abdominal aortic aneurysm, volume, aneurysm growth, natural history, EVAR surveillance, EVAR follow-up

## Abstract

There has been a debate about whether maximum diameter can be solely used to assess the natural history of abdominal aortic aneurysm. The aim of the present review is to collect all the available evidence on the role of abdominal aortic aneurysm (AAA) volume in the natural history of AAAs, including small untreated AAAs and AAAs treated by EVAR. The current literature appears to reinforce the role of volume as a supplementary measure for evaluating the natural history of AAA, in both intact AAAs and after EVAR. The clinical impact of AAA volume measurements remains unclear. Several studies show that volumetric analysis can assess changes in AAAs and predict successful endoluminal exclusion after EVAR more accurately than diameter. However, most studies lack strict standardized measurement criteria and well-defined outcome definitions. It remains unclear whether volumetry could replace diameter assessment in defining the risk of rupture of AAAs and identifying clinically relevant sac growth.

## 1. Introduction

Abdominal aortic aneurysm (AAA) maximum diameter has been used as the main characteristic to assess aneurysm growth and the need for repair [1]. Aneurysm diameter can be easily measured by ultrasound and constitutes a strong predictor of aortic rupture [2]. The increasing use of computed tomography, along with dedicated three-dimensional software, has achieved a more thorough evaluation of AAA geometry and, in particular, changes in the shape and size of AAAs.

The natural history of infrarenal abdominal aortic aneurysms includes their progressive growth, with a mean rate of 2.2 mm/year [1]. The larger the maximum diameter of an aneurysm, the larger its growth rate, and this has been calculated at 1.3 mm/year for 3 cm aneurysms and up to 3.6 mm/year for 5 cm aneurysms [1]. The decision to operate on a patient with an AAA is based on the equilibrium between the risk of aneurysm rupture and the hazard of operative mortality. According to current guidelines, the indication for elective repair of an asymptomatic infrarenal AAA is based solely on the maximum aortic diameter; however, the association between aneurysm volume and rupture risk has not been adequately studied [1]. There has been a debate about whether maximum diameter can be solely used to assess the need for an intervention [3]. Some studies support that diameter measurements cannot detect changes in shape in detail or tortuosity alterations of the aorta, and the existence of high rates of interobserver variability should also be acknowledged [4,5,6].

After endovascular aortic aneurysm repair (EVAR), AAA sac expansion (which has been reported in up to 8.5% of patients) represents not only treatment failure but also indicates a persistent risk of rupture despite the operation being performed [7]. Abdominal aortic aneurysm sac remodeling after EVAR has been considered an important factor that has been associated with the presence of endoleaks, graft migration, sac expansion, reintervention rate and long-term mortality [7,8]. Maximum aneurysm diameter measurements may overlook information about aortic remodeling and may also fail to detect morphology alterations at different levels of the aneurysm.

Aortic volume changes may constitute a more holistic assessment of AAA growth and successful exclusion after treatment. Also, meticulous assessment of small AAAs (4.0–5.5 cm) is crucial as the annual rupture rate of small AAAs is 2%, while some aneurysms with larger diameters above 5.5 cm remain stable [6]. In the case of EVAR, it is also important not only to find and define the parameters related to sac remodeling but to study the significance of each of these parameters on the short- and long-term outcomes after EVAR. The aim of the present study is to review all the available evidence on the role of AAA volume in AAA natural history, including small untreated AAAs and AAAs treated by EVAR.

## 2. Methods

**Figure 1:**
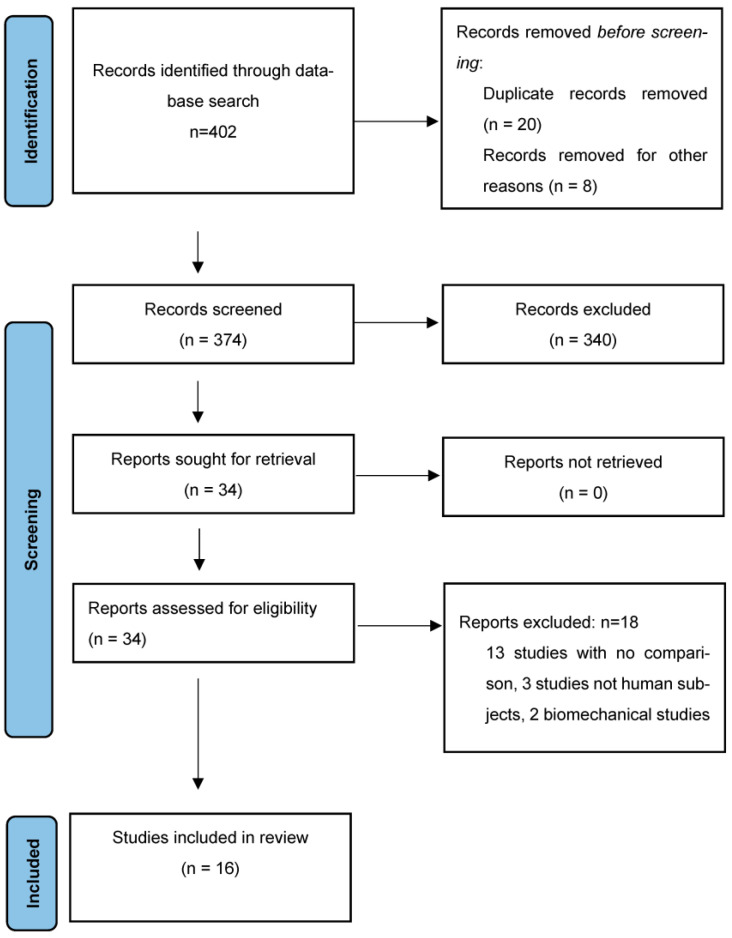
The Preferred Reporting Items for Systematic Reviews and Meta-Analyses (PRISMA) flowchart shows the selection process of this review.

Eligibility of studies: The Preferred Reporting Items for Systematic Reviews and Meta-Analyses (PRISMA) guidelines were followed (Figure 1). Observational studies of the English medical literature, from 2000 to 2023, reporting the role of AAA volume in AAA natural history and post-EVAR surveillance, were considered eligible and were included in the current review.

Search strategy: A systematic search of the English medical literature in the MEDLINE, Embase, and CENTRAL databases (via Ovid) was undertaken, in accordance with the PICO (Patient, Intervention, Comparison, Outcome model; Table 1), with an endpoint of 30 October 2023. The following search items, including expanding medical subject heading terms, were used in various combinations: (abdominal aortic aneurysm), (volume), (diameter), (sac expansion), (growth), (EVAR), (aneurysm surveillance). A consequent scrutiny was conducted after full-text assessment.

## 3. The Role of Volume in Small Abdominal Aortic Aneurysm Assessment

The monitoring of AAAs is mostly targeted to the sequential measurement of maximum abdominal aortic aneurysm diameter. Although a maximal aortic diameter of above 5.5 cm has been recognized to be associated with an increasing aneurysm rupture risk, studies have proven that up to 10% of AAAs with a diameter below 5 cm may still rupture [9]. Measuring aneurysm volume has also been proposed to possibly add to AAA assessment and improve growth prediction and help in treatment strategies. AAA growth differs in pace and dimensions in most patients [4]. Thus, if AAA growth occurs in a dimension other than maximum diameter, the measurement of the latter may not really reflect the actual sac enlargement, thereby underestimating the rupture risk.

Differences between diameter and volume have been reported in several studies assessing AAA natural history (Table 2). Parr et al. [10], investigating whether changes in aortic volume and diameter were similar in small AAAs, found that aortic volume changes are not always accompanied by comparable diameter changes. Nearly half the patients with sac growth did not have a consistent diameter increase. An enlargement of 1 mm in the orthogonal diameter was associated with a 4 cm^3^ increase in infrarenal aortic volume. Renapurkar et al. [11], investigating 100 patients with AAA, found that 64% of the patients had a significant change in sac volume but not in diameter (<2 mm) between studies. There was a modest correlation between diameter and volume change (r^2^ = 0.34; *p* = 0.001).

In addition, Ristl et al. [12] recently reported a significant association between the volume and maximum diameter in 84 patients with small AAAs. The average volume was approximately proportional to the average maximum diameter raised to a power of three. In all, 39% of the patients depicted similar growth rates for volume and maximum diameter, 33% showed a faster growth in volume, while in 27% of the cohort, maximum diameter growth was faster. At the intervention threshold of a maximum diameter of 55 mm, the median volume varied largely, from 103 to 167 mL. 

Olson et al. [13] investigated AAA volume growth by computed tomography (CT) in 250 patients during a 2-year follow-up period. The median and mean growth rates were 9.3 and 10.4%, respectively. A high baseline volume (regression coefficient 0.2, *p* < 0.001), tobacco use, tortuosity index (*p* < 0.001) and an absence of diabetes predicted volume growth. Interestingly, aneurysm tortuosity at baseline has been associated with a significantly larger annual volume growth rate (approximately 33 cm^3^/year).

Kontopodis et al. [14] investigated, in 34 patients, whether changes in intraluminal thrombus-related indicators, such as thrombus maximum thickness and volume, may precisely depict changes in aneurysm size during follow-up. Volume and maximum diameter growth rates correlated significantly (Spearman’s rho 0.6, *p* = 0.002). Rapid volumetric increase over time was linked with a 10× chance of needing an operation compared to those with a slower enlargement. A total of 12/15 of the surgically treated AAAs were in the high growth rate group (*p* = 0.005).

Spanos et al. [15] investigated different anatomical characteristics between intact and ruptured large abdominal aortic aneurysms (rAAAs > 80 mm). The total aneurysm volume was higher in ruptured (442 ± 140 mL) vs. intact AAAs (331 ± 143 mL, *p* = 0.014), while the intraluminal thrombus (ILT)/total aneurysm volume rate was lower in rAAAs vs. intact AAAs (55% vs. 70%, *p* = 0.02). The maximum diameter did not differ significantly between intact and ruptured AAAs (*p* = 0.150). The total aneurysm volume could predict rupture (AUC 0.68, *p* = 0.042). A threshold of total aneurysm volume <380 mL showed a 60% sensitivity and specificity, while maximum diameter could not predict rupture risk (AUC 0.62, *p* = 0.151).

Ghulam et al. [16] compared the diameter determined by ultrasound with aneurysm volume determined by three-dimensional ultrasound (3D-US) in 179 patients; 83% of the patients with an increasing aortic diameter depicted a similar volume increase. During a median follow-up of nearly one year, the mean increases in diameter and volume were 2.7 mm and 11.6 mL, respectively. AAAs with a stable diameter and a growing volume had higher rates of surgical repair than AAAs with a stable diameter and volume during follow-up. Khan et al. [17] examined whether duplex and three-dimensional tomographic ultrasound could recognize features associated with AAA growth in 128 patients on AAA surveillance. A stronger correlation of AAA growth with AAA volume than with diameter (r 0.46 vs. r 0.43, *p* < 0.01) was evident. With multivariate analysis, adding wall volume to diameter improved prediction of the growth rate (r^2^adjusted 0.22 vs. r^2^adjusted 0.18, *p* < 0.01).

## 4. The Role of Volume in Abdominal Aortic Aneurysm Surveillance after EVAR

EVAR has gained broad recognition as the treatment of choice in AAA patients with a favorable anatomy [18]. EVAR has been associated with lower 30-day mortality and morbidity and fewer complications than open repair, with a higher rate of risk of reintervention during follow-up [18,19]. Imaging surveillance after EVAR has been crucial in identifying graft-related complications and possibly averting rupture from persistent aneurysm sac enlargement. Thus far, the measurement of both maximum anteroposterior and transverse diameter has been considered as the gold standard for assessing sac shrinkage. Although successful aneurysm exclusion can be evaluated by maximum aneurysm diameter measurement, alterations in the aortic lumen and thrombus volume may play a significant supplementary role. Only a few studies have assessed the role of volume in AAA surveillance after EVAR (Table 3). Wever et al. [20] assessed the agreement between diameter and volume measurements after EVAR in 35 patients [4]. In 37% of the comparisons, discordance was found between the maximum diameter and volume measurements. The maximum diameter evaluation missed aneurysm shrinkage in 14% of the patients and aneurysm growth in 19% of the cases. Endoleak status and aneurysm growth did not correlate significantly. Volume increase and endoleaks had a stronger correlation (r = 0.37 at 6 months and r = 0.25 at 12 months) than maximum diameter and endoleaks (r = −0.07 and r = 0.11, respectively).

Kritparcha et al. [21] examined the sensitivity of three diameter measurements (maximum transverse/anteroposterior/in any orientation), determined from three-dimensional (3D) reconstructed computed tomography (CT), in identifying AAA growth after EVAR [5]. The diameter measurements did not detect sac growth in most of the cases. The agreement between the methods of diameter measurement and volume change was 35%, 15% and 25%, respectively, for a volume increase >10%, and 70%, 88% and 74%, respectively, for a volume decrease >10%. A total of 27% of the studies showed a significant volume increase and an unchanged maximum diameter [21]. Bargellini et al. [22] evaluated the volume alterations of 63 patients after EVAR. Patients with a higher preoperative maximum diameter showed a higher rate of endoleaks. Volume changes predicted endoleaks with an accuracy ranging between 74.6% and 84.1%, which was higher than that of alterations in diameter. A volume variation of <0.3% at 6 months has been the strongest independent predictor of endoleaks. Interestingly, one third of patients with endoleaks depicted no significant sac growth, while in 14% of the patients without endoleaks, the aneurysm sac was enlarged.

Schnitzbauer et al. [23] investigated the accuracy of different maximum diameter measurements compared with volume evaluation in 100 patients after EVAR. The authors used cutoff levels based on the reporting standards for aneurysm sac growth (diameter ≥ 5.0 mm, volume ≥ 5.0%). These levels had sensitivity/specificity rates for sac enlargement of 29%/95%, 33%/97%, 29%/99%, 33%/93% and 38%/96%, respectively, for the five maximum diameter values. Diameter evaluation failed to identify an aneurysm volume increase in 61–72% of patients with a persistent type II endoleak. Skrebunas et al. [24] discovered a moderate positive linear correlation between diameter and volume (R^2^ = 0.731) in 39 patients. In 28.2% of the patients, the diameter increased, while in 31% of the cohort, the volume increased. In eight of the eleven patients with the diameter increase, there were no clinical sequelae. In these patients, no significant difference between diameter and changes in sac volume were observed (*p* = 0.184).

Quan et al. [25] analyzed the relationship between the diameter and the volume measurement of the aorta after EVAR in 82 patients [9]. The growth rate of the aortic volume was significantly different from the increase rate of the maximum diameter. More endoleaks were evident in the aortic volume enlargement group than in the no-enlargement group (90.91% vs. 16.67%, *p* < 0.001). There was a higher secondary intervention rate in patients with volume growth. Frachin et al. [26] described the morphovolumetric changes of the AAA sac during follow-up after elective EVAR in 149 patients. Diameter shrinkage was detected in 18.1% and volume shrinkage in 28.2% of the patients. The AAA diameter shrinkage was associated with the preoperative diameter (*p* = 0.002). A persistent endoleak predicted the absence of volume shrinkage (*p* = 0.001; hazard ratio, 7.75; 95% CI, 2.282–26.291). The volume measurement showed a higher sensitivity than the measurement of the diameter in two dimensions.

Kargul et al. [27] investigated the association between maximum transverse diameter and volumetry in 51 EVAR patients, who underwent 59 reinterventions, and their connection with endoleaks. When measuring the maximum diameter, in 40/51 cases, there was a negative remodeling of the aneurysm sac. When using volume measurements, 48/51 patients had negative remodeling. Volumetry led to fewer missed negatives than maximum diameter (*p* < 0.001), constituting a more efficient screening tool for negative remodeling. Combining the diameter and volume measurements recognized 51 negative remodeling cases and eight positive changes and showed a higher sensitivity compared to maximum diameter alone.

## 5. The Role of Volume in Clinical Practice

The current literature appears to reinforce the role of volume as an additional criterion for assessing the natural history of AAAs, both intact and after EVAR. Certain advantages of volumetry should be considered. The ranges of AAA volumes are much wider than the corresponding AAA diameters and, therefore, absolute changes over time are higher. In addition, volume assessment seems to provide a more thorough evaluation of the AAA, since diameter values reveal the AAA’s size at only one site without taking into consideration changes at other sites (e.g., iliac arteries) or lengthening of the aorta. Remodeling of the aneurysm sac with a size increase below and above the area of maximum extension cannot be sufficiently evaluated by diameter assessment. On the other hand, the inclusion of volume assessment in regular AAA surveillance comes at the expense of augmented radiation exposure and contrast media as a CT scan is needed, compared to routine ultrasound surveillance. However, the progression of 3D ultrasonography has led to new opportunities in the noninvasive assessment of aneurysm volumes. A recent study showed that 3D contrast-enhanced ultrasound demonstrated good reproducibility and agreement with CTA in assessing total AAA volume and intraluminal thrombus volume and maximum thickness [28]. In addition, Lorenzen et al. have shown that analysis of AAA volume growth patterns with 3D-US is a practical and safe modality that seems more sensitive in detecting growth patterns than AAA diameter [29]. 

Currently, society guidelines recommend the maximum aortic diameter as the only solid indication for AAA treatment [1]. However, not all large aneurysms rupture, while sometimes small aneurysms may rupture, even with a maximum diameter < 5.5 cm [1,30]. In a recent study by our group, we found that although intraluminal thrombus volume was similar in patients with intact and ruptured aneurysms, the total aneurysm volume and the true lumen volume were larger in the latter [15]. Thus, AAAs with similar maximum diameters and different total aneurysm volumes proved to have a different rupture risk. This may suggest that diameter alone does not always specify the risk of rupture, and total aneurysm thrombus should be taken into consideration. 

Based on current guidelines, an AAA with a maximum diameter below 40 mm carries a low risk of rupture, while a threshold maximum diameter of 55 mm or greater indicates the need for repair [1,18]. Currently, there are no similar thresholds for aneurysm volumes. In a recent analysis of small AAAs, Olson et al. found that aneurysm volumes that reached the diameter repair threshold ranged from 102 cm^3^ to 142 cm^3^ in females and from 105 cm^3^ to 229 cm^3^ in males [13]. Interestingly, 30% of the females and 62% of the males with a diameter smaller than the repair threshold had volumes in the same respective ranges [13]. Similarly, in another study, the median volume varied significantly, from 103 to 167 mL [12]. This significant overlap of the volumes for patients above or below the repair diameter threshold really questions the potential of volume measurements to guide the need for intervention. Currently, a direct association between diameter thresholds and volume and rupture risk is not feasible. More data are needed to recognize small-diameter and high-volume AAAs with a high risk of rupture.

Current ESVS guidelines recommend that patients at a low risk of EVAR failure, based on the first postoperative CTA, may be considered for less-frequent surveillance imaging [1]. However, this recommendation considers the maximum diameter alterations and the presence of an endoleak as predictors of a stricter surveillance protocol. Franchin et al. found that an absence of volume shrinkage correlated most with unfavorable outcomes [26]. As there are no robust data to change the time interval between follow-up CTAs, more studies are needed to confirm the significant value of volume surveillance after EVAR and its association with a worse outcome.

Other factors like aortic wall tension measurements have been used for predicting AAA rupture. The average peak wall tension has been found to be nearly 45% higher in ruptured than in intact AAAs, even though the former were nearly 55% larger in diameter [31]. A recent study compared the peak wall stress (PWS) and peak wall rupture index (PWRI) in ruptured and intact AAAs; the sensitivity analysis found that patients with a high PWS were five times more likely to sustain a rupture at both low and high blood pressure levels [32]. However, PWS values have been based on small-scale studies and have not been adequately standardized with an accurate measurement methodology. In the future, aortic wall tension measurements could act as surrogate markers of rupture risk, although more studies are needed to validate their use in daily clinical practice.

Volume measurements have been shown to be reproducible and reliable, showing excellent interreader agreement, with a repeatability coefficient < 6 mL and <6% for relative volume growth [33]. One of the main concerns of volume assessment in daily clinical practice has been the potential lack of time efficiency. The general use of volumetry has been crucially hampered by the time required for its implementation. In the last decade, 3D semiautomatic software has provided quick, easy and reliable platforms for applying volumetry in daily practice, reducing the time for volumetry from 45 min to 15 min [34]. Furthermore, recently, an innovative, fully automated software (PRAEVAorta version 2; Nurea, Bordeaux, France) using artificial intelligence has emerged as an additional tool for volume assessment. Caradu et al., using this software, found that the mean volumetric similarity reached 0.873 ± 0.100 for the lumen and 0.903 ± 0.091 for the thrombus [35]. The segmentation time was nine times faster with the fully automatic method (2.5 min vs. 22 min per patient with the manually corrected method; *p* < 0.0001). New technologies may ease the universal implementation of volumetry in daily clinical practice and act as decision-making tools for the diagnosis and follow-up of AAAs.

## 6. Conclusions

The clinical impact of AAA volume measurement remains unclear. Several studies show that volumetric analysis can assess changes in AAA and predict successful endoluminal exclusion after EVAR more accurately than diameter. However, most studies lack strict standardized measurement criteria and well-defined outcome definitions. It remains unclear whether volumetry could replace diameter assessment in defining the risk of rupture of an AAA and identifying clinically relevant sac growth.

## Figures and Tables

**Table 1:** The PICO (patient, intervention, comparison, outcome) model was used to define the clinical questions and clinically relevant evidence in the literature in this systematic review evaluating the role of AAA volume in the natural history of AAA and post-EVAR surveillance.

P Patient, population or problem	Patients with abdominal aortic aneurysm
I Intervention, prognostic factor or exposure	AAAs intact or post-EVAR
C Comparison of intervention	Sac volume vs. diameter measurements
O Outcome you would like to measure or achieve	Aneurysm sac volume
What type of question are you asking?	What is the role of sac volume in AAA surveillance?
Type of study you want to find	Observational studies reporting the value of sac volume in AAA surveillance compared to diameter measurement

**Table 2:** Studies on the role of volume in abdominal aortic aneurysm surveillance.

Authors	Year	Patients	Follow-Up	Results
Ristl et al. [10]	2023	84	NA	A total of 39% of patients depicted similar growth rates for volume and maximum diameter. In all, 33% of patients showed a faster growth in volume, while in 27% of the cohort, growth was faster in maximum diameter.
Parr et al. [11]	2013	57	1.7 years (1.1–3.3 years)	In total, 42% of patients with an increased aortic volume above the 95% limit of agreement did not show relevant axial or orthogonal diameter changes.
Renapurkar et al. [12]	2012	100	at least 6 months	A modest association between diameter change and volume change was evident (r^2^ = 0.34; *p* = 0.001). Sixty-four patients had no measurable change in maximal diameter (≤2 mm) between studies, while volume varied widely (−2 to 69 mL).
Olson et al. [13]	2022	250	6 months to 2 years	Baseline volume and maximum diameter accounted for 30% and 13% of volume growth variance subsequently. A high baseline volume (regression coefficient 0.2, *p* < 0.001), tobacco use, tortuosity index (*p* < 0.001) and an absence of diabetes predicted volume growth. Volumes of AAAs that reached the repair threshold varied significantly (102 cm^3^–142 cm^3^ in female patients and 105 cm^3^–229 cm^3^ in male patients)
Kontopodis et al. [14]	2014	34	median follow-up 12 months (6–36 months)	A rapid increase in volumetry over time was linked with a 10× chance of needing an operation, compared to those with a slower enlargement. In total, 12/15 of the surgically treated AAAs were in the high growth rate group (*p* = 0.005).
Spanos et al. [15]	2020	62	NA	Maximum diameter did not differ significantly between intact and ruptured AAAs (*p* = 0.150). Total aneurysm volume could predict rupture (AUC 0.68, *p* = 0.042). A threshold of total aneurysm volume < 380 mL showed a 60% sensitivity and specificity. Maximum diameter could not predict rupture risk (AUC 0.62, *p* = 0.151).
Ghulam et al. [16]	2017	179	1 year	During a median follow-up of nearly one year, mean increase in diameter and volume were 2.7 mm and 11.6 mL, respectively. AAAs with a stable diameter and a growing volume had higher rates of surgical repair than AAAs with a stable diameter and volume during follow-up.
Khan et al. [17]	2022	128	at least 2 years	A stronger correlation of AAA growth with AAA volume than with diameter (r = 0.46 vs. r = 0.43, *p* < 0.01) was evident. With multivariate analysis, adding wall volume to diameter improved prediction of the growth rate (r^2^adjusted 0.22 vs. r^2^adjusted 0.18, *p* < 0.01).

**Table 3:** Studies on the role of volume in abdominal aortic aneurysm surveillance after EVAR.

Authors	Year	Patients	Follow-Up	Results
Wever et al. [20]	2000	35	6–48 months	Maximum diameter evaluation missed aneurysm shrinkage in 14% of patients and aneurysm growth in 19% of cases Endoleak status and aneurysm growth did not correlate significantly. Volume increase and endoleaks correlated stronger (r = 0.37 at 6 months, r = 0.25 at 12 months) than maximum diameter and endoleaks (r = −0.07 and r = 0.11, respectively)
Kritparcha et al. [21]	2004	68	mean 12.7 months (range 6–36 months)	Agreement between methods of diameter measurement (maximum transverse, maximum anteroposterior and maximum in any orientation) and volume change were 35%, 15% and 25% for volume increase >10%, respectively, and 70%, 88% and 74%, respectively, for volume decrease >10% 27% of the studies showed significant volume increase and unchanged maximum diameter
Bargellini et al. [22]	2005	63	6–24 months	Volume changes predicted endoleaks with an accuracy ranging between 74.6% and 84.1%, which was higher than that of diameter alterations A volume change at 6 months of <0.3% has been the strongest independent predictor of endoleaks
Schnitzbauer et al. [23]	2017	100	359 ± 333 days	Diameter evaluation failed to identify aneurysm volume increase in 61–72% of patients with a persistent type II endoleak.
Skrebunas et al. [24]	2019	39	635.3 ± 249.7 days	A moderate positive linear correlation between diameter and volume (r^2^ = 0.731)
Quan et al. [25]	2019	82	21.02 ± 17.59	The growth rate of aortic volume was significantly different from the increase rate of maximum diameter More endoleaks were evident in the aortic volume enlargement group than in the no-enlargement group (90.91% vs. 16.67%, *p* < 0.001).
Frachin et al. [26]	2021	149	42 months (22.5–58 months)	Diameter shrinkage was detected in 18.1%, volume shrinkage in 28.2% A persistent endoleak predicted the absence of volume shrinkage (*p* = 0.001; hazard ratio, 7.75; 95% CI, 2.282–26.291) Volume measurement showed a higher sensitivity than the measurement of the diameter in two dimensions
Kargul et al. [27]	2023	51	NA	When measuring the maximum diameter, in 40/51 cases, there was a negative remodeling of the aneurysm sac. When using volume measurements, 48/51 patients had negative remodeling Sac volumetry does not seem to characterize the endoleak type in detail

## Data Availability

No new data were created or analyzed in this study. Data sharing is not applicable to this article.
